# Anti-COVID Vaccination for Adolescents: A Survey on Determinants of Vaccine Parental Hesitancy

**DOI:** 10.3390/vaccines9111309

**Published:** 2021-11-10

**Authors:** Stefano Zona, Simonetta Partesotti, Andrea Bergomi, Cristiano Rosafio, Francesco Antodaro, Susanna Esposito

**Affiliations:** 1 Primary Health Care Department, Local Health Agency of Modena, 41121 Modena, Italy; si.partesotti@ausl.mo.it (S.P.); susannamariaroberta.esposito@unipr.it (S.E.); 2 Primary Care Pediatricians, Local Health Agency of Modena, 41121 Modena, Italy; nao.bergomi@gmail.com (A.B.); cristiano.rosafio@gmail.com (C.R.); francesco.antodaro@gmail.com (F.A.); 3 Pediatric Clinic, Pietro Barilla Children’s Hospital, University of Parma, 43126 Parma, Italy

**Keywords:** adolescents, COVID-19, COVID-19 vaccines, pediatric infectious diseases, vaccine hesitancy

## Abstract

Vaccine hesitancy has been considered one of the most severe threats to global health, as it represents an obstacle to achieving adequate vaccination coverage. Recent research studies aimed at investigating the propensity for anti-COVID vaccination among adults have found a high prevalence of vaccine hesitancy, but few data are available on parental vaccine hesitancy. We therefore built an anonymous online survey to investigate the factors related to the vaccine hesitancy of parents of adolescents between 12 and 17 years of age, with a special focus on demographic factors and the domains of confidence and complacency. The online survey was conducted by using the Crowd Signal platform from 15 July to 16 August 2021, in Italy. A total of 1799 analyzable questionnaires were analyzed. Overall, Favorable and Doubtful parents declared a higher level of confidence on safety and efficacy of pediatric vaccines and on confidence in health institutions than Hesitant/Reluctant ones (*p*-values < 0.001). The univariate multinomial logistic regression analysis and the multivariate multinomial logistic regression analysis showed that the Hesitant/Reluctant parents were younger than 40 years of age, with a secondary-school or three-year degree, free-lance, with a family income below €28,000, with an erroneous perception of the risk of COVID-19 as disease and with fear of anti-COVID vaccination. These results, which should be confirmed in a larger population and in different geographical areas, should lead Institutions and stakeholders to identify targeted communication tools to improve trust in health institutions, especially by younger parents.

## 1. Background

After the discovery in Wuhan, China, of the human-to-human transmission of a new beta-coronavirus (SARS-CoV-2) [1], the World Health Organization (WHO) has declared the pandemic spread of the virus that causes the disease called coronavirus disease 2019 (COVID-19) [2]. The pediatric population has suffered both direct and indirect effects of the pandemic, with the indirect ones being primary due to the closure of schools and the interruption of sports and social activities [3].

Since December 2020, the European Medicines Agency (EMA) has progressively conditionally authorized the first anti-COVID vaccines, of which two with mRNA (Comirnaty [4], produced by Pfizer/BioNTech, initially indicated for subjects ≥16 years of age; and Spikevax [5], produced by Moderna, initially recommended for subjects ≥18 years old) and two with a non-replicating adenoviral vector (Vaxzevria, produced by AstraZeneca/Oxford [6], and COVID-19 Vaccine Janssen [7], produced by Johnson & Johnson, both recommended for subjects aged ≥18 years). In Italy, the vaccination campaign began in January 2021, and it was conducted by identifying target populations [8] (old elderly and “fragile” individuals presenting risk factors for severe COVID-19 disease, healthcare providers and law enforcement) and then progressively opening to the whole population. In May 2021, EMA authorized the use of Comirnaty for subjects ≥12 years of age [9]; in July 2021, the Spikevax vaccine was also authorized for subjects ≥12 years of age [10].

Despite the chance of having access to vaccination free of charge, according to public data from the Local Health Agency of Modena, Italy, in June 2021 only one-third of adolescents 12–19 years old had had access to vaccination or made a reservation yet [11], as a result of parental vaccine hesitancy towards anti-COVID vaccination. In October 2022, this percentage increased to two-thirds, showing that there is still a long way to go to reach adequate vaccination coverage [11]. Studies on vaccination hesitancy have shown that vaccine hesitancy is due to a complex of factors, which can vary in different cultures and over time. In 2015, the SAGE Working Group on Vaccine Hesitancy of the WHO [12] proposed an interpretative model of vaccination hesitancy, consisting of “3Cs”: confidence, which groups together the determinants related to trust in vaccination practice and institutions sanitary; convenience, which groups together the determinants related to the chance of receiving vaccination due to the real or perceived quality of the health services in charge (i.e., free access to vaccination, proximity to the center where the vaccination is administered, the chance of obtaining understandable information in one’s own language on vaccines and recommendations); and complacency, which identifies the cultural and individual determinants inducing a poor perception of the risks of vaccine preventable diseases.

Vaccine hesitancy, even in the pre-pandemic era [13], has been considered one of the most severe threats to global health, as it represents an obstacle to achieving vaccination coverage beyond the safety thresholds useful for reducing infectious diseases’ burden and overloading health services. Vaccine hesitancy towards anti-COVID vaccination could become one of the causes of the perpetuation of the spread of the SARS-CoV-2 pandemic [14,15,16]. Considering the most recent SARS-CoV-2 R0 data, estimated to about six (i.e., an infected person transmits the infection on average to six other subjects) also due to the spread of the Delta variant [17,18], the achievement of so-called “community immunity” or “herd immunity” is unlikely if younger people are not appropriately covered by vaccination. There are two fundamental reasons for extending vaccination to adolescents: to establish herd immunity, it is necessary that the share of immunes be uniformly distributed in the population [19]; and the social contacts of adolescents are very frequent [20].

Recent research studies aimed at investigating the propensity for anti-COVID vaccination among adults have found a high prevalence of vaccine hesitancy [21,22,23,24,25], mainly linked to concerns about the secondary effects and adverse events associated with vaccines, as well as their rapid authorization. Moreover, a relevant factor seems to be the distrust of governments and health institutions [24,26]. Few data are available on parental vaccine hesitancy towards anti-COVID vaccination, and a large majority of these data were collected when vaccines against COVID were not authorized for use in pediatric-age subjects [27,28,29,30,31]. We therefore built an anonymous online survey to investigate the factors related to the parental vaccine hesitancy of parents of adolescents between 12 and 17 years of age, with a special focus on demographic factors and the domains of confidence and complacency.

## 2. Methods

A questionnaire on parental vaccine hesitancy was developed by pediatricians and infectious diseases specialists (SZ, SP and AB) of the Local Health Agency of Modena, Italy. Before administration, a pilot study was conducted: it was administered twice with a one-week interval to a convenience sample of 3 pediatricians and 12 parents. Moreover, it was translated in English and French by mother-tongue cultural mediators. The online survey was collected by using the Crowd Signal platform (www.crowdsignal.com, accessed on 1 November 2021) from 15 July to 16 August 2021, with restriction from the same device to reduce the risk of repetition by the same user. The link to the survey was distributed through the Local Health Agency of Modena, Italy (Facebook page and website), and with the voluntary and free collaboration of general practitioners and primary-care pediatricians. 

Overall, 4632 parents of children 12–17 years old received the questionnaire. Answers of participants were unified in a unique database from the questionnaire in Italian (n = 4625), in English (n = 4) and in French (n = 3). Answers with completion times below 4 min were discarded a priori, for a total of 2453 questionnaires. Of the remaining 2179 questionnaires, additional 338 questionnaires were excluded from the analysis, as they lacked one or more answers to section 4, used to define clustering in the “Favorable”, “Doubtful” and “Hesitant/Reluctant” groups. A further 42 questionnaires were excluded as implausible (i.e., age declared too young or too old to have a child aged 12–17 years; number of children declared implausible), thus arriving at a total of 1799 analyzable questionnaires (38.9%).

The survey consisted of 5 sections (see Appendix A):

 **-** Collection of demographic data (i.e., age, gender, area of residence, educational qualification, occupation, annual income of the family unit, number of children, number of children between 12 and 17 years of age, nationality);
 **-** Acceptability and general vaccine in the pre-pandemic era;
 **-** Perception of the risk of COVID-19 disease;
 **-** Perception of the safety and efficacy of anti-COVID vaccines;
 **-** Propensity for anti-COVID vaccination for oneself and for children.

The 3 clusters (i.e., “Favorable”, “Doubtful” and “Hesitant/Reluctant”) were defined by using the method of grouping by median (k-medians) with Euclidean distance of the answers to the question Q21:

 **-** I had my children vaccinated/I will definitely have my children vaccinated;
 **-** I have no intention of vaccinating my children;
 **-** The vaccine has an excellent level of safety;
 **-** The vaccine has not been sufficiently tested;
 **-** I want to wait before vaccinating;
 **-** My children are healthy, so they do not need vaccines;
 **-** I have vaccinated/will vaccinate my children because they are vulnerable to pathological conditions.

Information Scores were also constructed based on the agreement of some statements. The Information Score on pediatric vaccines was constructed with the arithmetic mean of the following scores:

- Q10.S1. Pediatric vaccines are useful for preventing life-threatening diseases (3 for values ≥4; 2 for values equal to 3; 1 for values ≤2);
- Q10.S2. Pediatric vaccines have saved millions of lives since they were invented (3 for values ≥4; 2 for values equal to 3; 1 for values ≤2);
- Q10.S3. Pediatric vaccines are poorly studied (1 for values ≥4; 2 for values equal to 3; 3 for values ≤2);
- Q10.S4. Pediatric vaccines often lead to serious adverse events (1 for values ≥4; 2 for values equal to 3; 3 for values ≤2);
- Q10.S6. Vaccinating children is important to stop some epidemics (i.e., measles, rubella, pertussis and meningococcal meningitis) (3 for values ≥4; 2 for values equal to 3; 1 for values ≤2).

The Information Score on COVID-19 was constructed with the arithmetic mean of the following scores:

- Q13.S2. COVID-19 is a normal influenza or little more (1 for values ≥4; 2 for values equal to 3; 3 for values ≤2);
- Q13.S4. The pandemic has deteriorated the possibilities of prevention and treatment by the health system (3 for values ≥4; 2 for values equal to 3; 1 for values ≤2);
- Q13.S7. Children and young people, even if they get sick, never have COVID-19-related problems (1 for values ≥4; 2 for values equal to 3; 3 for values ≤2);
- Q13.S8. COVID-19 is dangerous only for the elderly and those with pathologies (1 for values ≥4; 2 for values equal to 3; 3 for values ≤2).

The Information Score on anti-COVID vaccines was constructed with the arithmetic mean of the following scores:

- Q17.S1. Authorized anti-COVID vaccines are still experimental (1 for values ≥4; 2 for values equal to 3; 3 for values ≤2);
- Q17.S2. Many of the dangerous effects on the health of the recipient of the anti-COVID vaccines are not known (1 for values ≥4; 2 for values equal to 3; 3 for values ≤2);
- Q17.S4. Anti-COVID vaccines are useless to contain the spread of the virus (1 for values ≥4; 2 for values equal to 3; 3 for values ≤2);
- Q17.S5. Anti-COVID vaccines will give rise to dangerous variants of the virus (1 for values ≥4; 2 for values equal to 3; 3 for values ≤2);
- Q17.S7. Anti-COVID vaccines are the best way to avoid deaths and hospitalizations (3 for values ≥4; 2 for values equal to 3; 1 for values ≤2);

Similarly, a Fear Score of vaccines was constructed by using the scores on the following statements:

- Q11.S2. When I receive invitations for vaccinations, I start to feel anxious (3 for values ≥4; 2 for values equal to 3; 1 for values ≤2);
- Q11.S3. I trust the institutions; the proposed vaccines are the best choice for my children (1 for values ≥4; 2 for values equal to 3; 3 for values ≤2);
- Q11.S6. After vaccination, I am afraid of serious adverse events (3 for values ≥4; 2 for values equal to 3; 1 for values ≤2).

The demographic differences between the groups were analyzed with parametric methods (ANOVA) for continuous variables with normal distribution and non-parametric (Kruskal–Wallis test) for continuous variables with non-normal distribution. Categorical variables were analyzed by using the Χ^2^ test or Fisher’s exact test. The scores relating to the statements placed in the various sections of the questionnaire and relating to the Information Scores were represented with box plots; differences in medians were analyzed using the Kruskal–Wallis test. For questions with categorical answers, the *Χ^2^* test or Fisher’s exact test was used. In the case of statistical significance reached, a post hoc analysis with Bonferroni adjustment was performed.

Finally, a univariate and subsequently multivariate model of multinomial logistic regression was constructed, chosen from among different models through the Akaike information criterion (AIC), to identify potential factors associated with the risk of vaccination doubt or hesitancy of parents against anti-COVID vaccination for their sons. Statistical significance was considered for *p*-values <0.05. Based on the results of the univariate multinomial analyzes, several multivariate multinomial logistic regression models were constructed, in which the following independent variables were inserted:

- Model A: age, gender, work situation, pre-pandemic hesitancy, experience of the death of a loved one due to COVID-19;
- Model B: age, gender, work situation, Information Score on pediatric vaccines, experience of the death of a loved one due to COVID-19;
- Model C: age, gender, work situation, Information Score on pediatric vaccines, Information Score on COVID-19;
- Model D: age, gender, work situation, Information Score on pediatric vaccines, Information Score on COVID-19, experience of the death of a loved one due to COVID-19;
- Model E: age, gender, work situation, Fear Score, experience of the death of a loved one due to COVID-19;
- Model F: age, gender, work situation, Information Score on COVID-19, Fear Score, experience of the death of a loved one due to COVID-19;
- Model G: age, gender, work situation, Information Score on COVID-19, Fear Score;
- Model H: age, gender, work situation, Information Score on pediatric vaccines, Information Score on COVID-19, Fear Score;
- Model I: age, gender, educational qualification, Information Score on pediatric vaccines, Information Score on COVID-19, Fear Score;
- Model J: age, gender, work situation, educational qualification, Information Score on pediatric vaccines, Information Score on COVID-19, Fear Score;
- Model K: age, gender, work situation, educational qualification, sources of information, Fear Score.

All statistical analyzes were conducted with the STATA 13.1 software package for Mac (Stata-Corp, College Station, TX, USA). Being a descriptive study, it was not performed a formal calculation of the sample size.

## 3. Results

Out of a total of 1799 analyzable questionnaires, 1303 were filled in by females (72.4%), 424 were filled in by men (23.6%), 65 questionnaires did not indicate gender (3.6%) and six indicated non-binary gender (0.3%). Table 1 summarizes the demographic characteristics of those who responded to the questionnaire. The declared average age was 45 years (±5.8). The majority of the questionnaires was received from Northern Italy, from the provinces of Modena (642, 37.6%), Monza and Brianza (126, 7.4%) and Bologna (81, 4.7%), in particular. A majority of people stated that they were in permanent employment (1044, 58.0%) or self-employed (375, 20.8%). Regarding education level, high-school graduates were 704 (39.1%), and 542 (30.1%) declared a master’s degree. The number of children reported was one child for 465 questionnaires (25.8%), two children for 955 (53.1%) and three or more children for 379 (21.1%). Overall, 1296 (72.0%) said they had a child between the ages of 12 and 17 years, while 503 (27.9%) said they were the parent of more than one child between the ages of 12 and 17 years. Regarding the declared family income, the majority declared an income >28,000 euros/year. Italians represented the vast majority of those who answered the questionnaires (1720, 95.6%); 41 (2.3%) did not declare ethnicity, 14 (0.8%) declared to come from Western Europe, seven (0.4%) from Eastern Europe, nine (0.50%) from North Africa, one (0.06%) from Central Africa, four (0.2%) from Asia and three (0.2%) from the Americas. Using the cluster analysis based on the level of agreement of the statements with respect to the propensity of the anti-COVID vaccination of adolescents, three groups were generated: “Favorable” (477, 26.5%), “Doubtful” (526, 29.2%) and “Hesitant/Reluctant” (796, 44.2%). The three groups differed for age, with a higher prevalence of youngest in the Hesitant/Reluctant group; for gender, with women being more prevalent in the Doubtful group; for work condition, with self-employed and non-answering being more present in the Hesitant/Reluctant group; and for declared annual income, with the poorest being more prevalent in the Hesitant/Reluctant group. After Bonferroni adjustment, we did not find significant difference between Favorable and Doubtful groups for education level, number of sons (including number of sons 12–17 years old), nationality and residence; Hesitant/Reluctant differed from other groups principally for the no-answer option in education level (although master’s degree was less represented than in other groups), in nationality and in residence.

**Figure 1:**
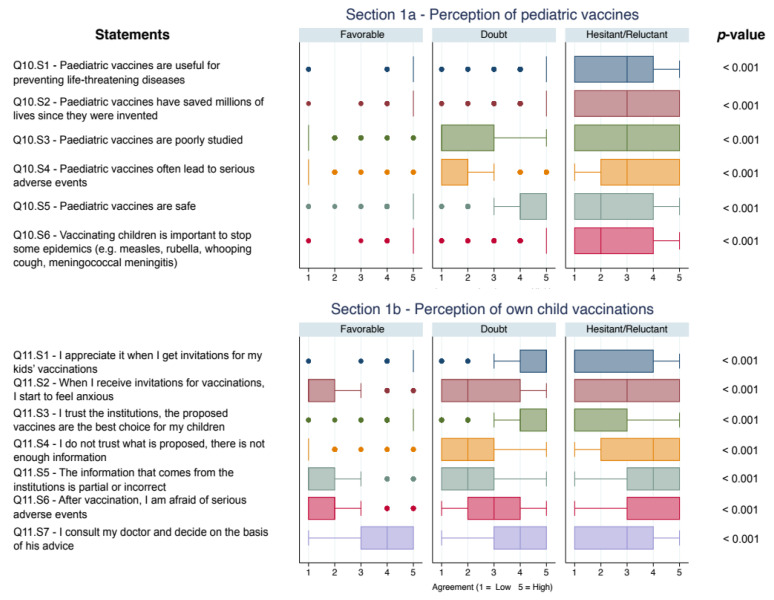
Boxplot of concordance scores on confidence on vaccines and vaccinations. All *p*-values < 0.05 after Bonferroni adjustment.

Figure 1 shows the concordance scores, expressed via boxplot (median and interquartile range), to the statements proposed in Q10. Overall, Favorable and Doubtful parents declared a higher level of confidence on safety and efficacy of pediatric vaccines than Hesitant/Reluctant, investigated through statements Q10.S1 and Q10.S5 (all *p*-values < 0.001). Similar results were reported for statements included in Q11, which investigated confidence in health institutions in relationship to the personal experience of own child vaccination through statements Q11.S3 and Q11.S3 (all *p*-values < 0.001).

The results of the following question are shown below:

- Q12 Did all of your children have pediatric vaccinations according to the recommended schedule?

They answered “Yes” in 1351 (75.1%); “No, but for postponements due to health reasons” in 64 (3.6%); “No, because I did not have sufficient reassurance” in 186 (10.3%); and they did not answer in 198 (11.0%). Therefore, only those who answered “No because I did not have sufficient reassurance” were considered as Hesitant: three of them were identified in the “Favorable” group (equal to 0.6%), while one did not answer; five in the “Doubtful” group (0.9%), while 16 (3.0%) did not answer; in the “Hesitant/Reluctant” group, 177 (22.2%) answered that they did not respect the recommended vaccine schedule and 187 (23.4%) did not answer; difference among groups were calculated with Fisher’s exact test (*p* < 0.001) and confirmed after Bonferroni adjustment.

**Figure 2:**
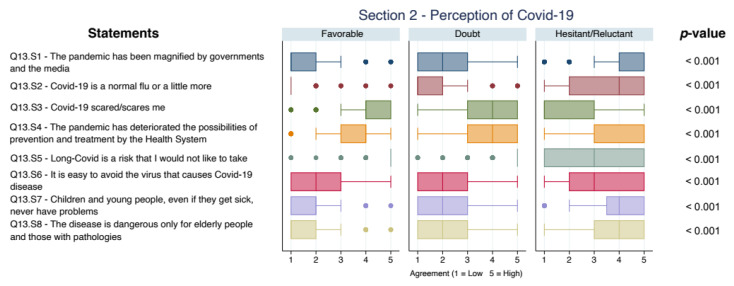
Boxplot of concordance scores on perception of COVID-19. All *p*-values < 0.05 after Bonferroni adjustment.

Figure 2 describes the scores on the statements regarding the risk perception of COVID-19 in the different groups. Risk perception of COVID-19 differed in groups: Hesitant/Reluctant parents declared a higher level of complacency both for themselves and for children than Favorable and Doubtful ones. In particular, the median score for the statement Q13.S7 was 2 (IQR 1; 2) for Favorable ones, 2 (IQR 1; 3) for Doubtful and 4 (IQR 3.5; 5) for Hesitant/Reluctant (Kruskal–Wallis test *p* < 0.001, difference among groups were confirmed after Bonferroni adjustment).

Overall, 1077 (59.8%) answered that they or a household had a positive swab, only 77 (4.3%) did not respond, 766 (42.6%) answered that they or a loved one had symptomatic disease and 66 (3.7%) did not respond. The results for the following question are presented below:

- Q16. Has a family member or loved one died due to COVID-19?

In total, 311 (17.3%) answered affirmatively, and 66 (3.7%) did not answer. Proportions of answers of Favorable and Doubtful parents did not significantly differ, while Hesitant/Reluctant ones were more likely not to answer, after Bonferroni correction.

Overall, Doubtful and Hesitant/Reluctant appeared less well informed than Favorable ones, according to median scores to the statement Q17.S1, which resulted in 2 (IQR 1; 3), 3 (3; 5) and 5 (5; 5) for Favorable, Doubtful and Hesitant/Reluctant, respectively, with *p* < 0.001; to the statement Q17.S5, median scores resulted in being 1 (IQR 1; 1), 1 (1; 2) and 5 (3; 5) for Favorable, Doubtful and Hesitant/Reluctant ones, respectively (*p* < 0.001). Regarding information sources, Favorable and Doubtful were more likely to consider general practitioners as authoritative (Q18.S2): median scores were 4 (IQR 3; 5), 4 (3; 5) and 3 (1; 4) for Favorable, Doubtful and Hesitant/Reluctant, respectively, with *p* < 0.001. Similar results were found for doctors working for the Health National System (Q18.S2): 5 (IQR 3; 5), 4 (3; 5) and 3 (1; 4) for Favorable, Doubtful and Hesitant/Reluctant, respectively, with *p* < 0.001.

**Figure 3:**
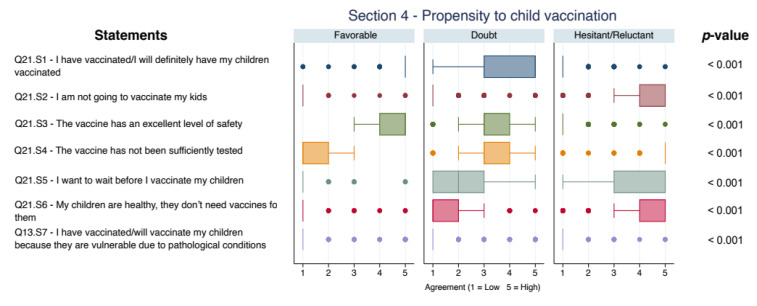
Boxplot of concordance scores on risk perception, safety and propensity for anti-COVID vaccination for adolescents. All *p*-values < 0.05 after Bonferroni adjustment (excluded Q13.S7: no significant difference was found between Favorable and Doubtful, *p* = 0.15).

The comparison analysis of risk perception, safety and propensity for anti-COVID vaccination for adolescents is described in Figure 3.

Regarding individual adult vaccination hesitancy, Favorable and Doubtful parents received or booked significantly more often anti-COVID vaccination (475/476, 99.6%, and 505/507, 96.0%, vs. 174/313, 21.9%), as well as recommended the anti-COVID vaccination (472/476, 98.9%, and 450/510, 85.6%, vs. 174/231, 14.1%) than the Hesitant/Reluctant ones (*p* < 0.001 for all the comparisons).

**Figure 4:**
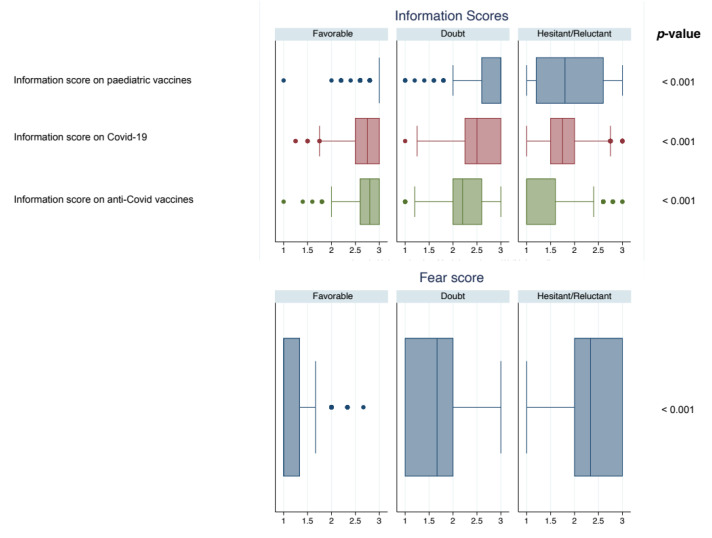
Boxplot of the Information Scores and the Fear Score. All *p*-values < 0.05 after Bonferroni adjustment.

Figure 4 shows the comparison analysis of the Information Scores and the Fear Score. Hesitant/Reluctant showed lower median scores than Doubtful and Favorable on pediatric vaccines: 1.8 (IQR 1.2; 2.6), 3 (2.6; 3) and 3 (3; 3), respectively, with *p* < 0.001. Similar results were found for Information Score on COVID-19: 1.75 (IQR 1.5; 2) for Hesitant/Reluctant, 2.5 (2.25; 3) for Doubtful and 2.75 (2.5; 3) for Favorable, with *p* < 0.001. The Information Score on anti-COVID vaccines showed the same trend: 1 (IQR 1; 1.6) for Hesitant/Reluctant, 2.2 (2.0; 2.6) for Doubtful and for 2.8 (2.6; 3) Favorable, with *p* < 0.001.

The univariate multinomial logistic regression analysis is shown in Table 2. An age younger than 50 years was significantly associated with being Doubtful (*p* = 0.026), while an age less than 40 years was associated with being Hesitant/Reluctant (*p* < 0.001). The female gender resulted in being associated with Doubtful (*p* = 0.002); the male gender and no answer/non-binary resulted associated with Hesitant/Reluctant (*p* < 0.001). People declaring a Master’s degree or PhD were less likely to be Doubtful (*p* = 0.031 and *p* = 0.018, respectively), while Hesitancy resulted associated with “no answer” (*p* = 0.010) and negatively associated with MA (*p* = 0.015). No-work condition resulted in being associated with Doubtful, while self-employed (RRR 2.23, *p* < 0.001) and retired/no answer (RR = 8.75, *p* < 0.001) resulted in being associated with Hesitant/Reluctant. A declared annual income less than 28,000€ (*p* = 0.001) and “no answer” (*p* < 0.001) were associated with Hesitant/Reluctant; “no answer” resulted in being associated with being Doubtful (RR = 2.08, *p* < 0.001).

Based on AIC described in the Methods, the Model H was identified as the most parsimonious and most reliable (data not shown). Table 3 shows the results of multivariate multinomial logistic regression analysis.

After multivariate multinomial logistic regression analysis, Doubtful resulted negatively associated with age more than 50 years (*p* = 0.028), male gender (*p* = 0.036), unemployment/unpaid domestic work (*p* = 0.002), Information Score on pediatric vaccines (*p* < 0.001), Information Score on COVID-19 (*p* < 0.001), and positively associated with Fear Score (*p* < 0.001). Hesitant/Reluctant resulted negatively associated with older age (*p* < 0.001 both for 41–50 years old and more than 50 years old), with unemployment/unpaid domestic work (*p* = 0.003), with Information Score on pediatric vaccines (*p* < 0.001) and on COVID-19 (*p* < 0.001), while it resulted in being positively associated with self-employment (*p* = 0.044), retired/no answer (*p* = 0.003) and Fear Score (*p* < 0.001).

## 4. Discussion

The commercialization of anti-COVID vaccines has brought the issue of vaccine safety and efficacy back into public debate. In Italy, the vaccine parental hesitancy of pediatric vaccines had an impact at the beginning of the second decade of the 2000s, when vaccination coverage dropped to alert levels [32]. In 2017, after the introduction of law 119/17 on the obligation to vaccinate to access educational services for 0–6 years, the phenomenon of vaccination hesitancy appeared to decrease [33]. According to a poll conducted by CENSIS, it found that vaccination hesitancy made up of “doubters” was about 11% of Italian parents, while ideological reluctance was reduced to less than 1% of parents [34]. From May 2021, i.e., from the first conditional authorization by EMA of the Pfizer/BioNTech anti-COVID vaccine for adolescents ≥12 years old, the theme of vaccine hesitancy seemed to have reappeared: in fact, in the province of Modena, Italy, on June 30, less than 40% of adolescents between 12 and 17 years of age had entered the vaccination course against COVID (complete, incomplete vaccination or reservation) [35].

In our survey aimed at the parents of adolescents between 12 and 17 years of age, vaccine hesitancy was mainly determined by three aspects: “trust” in vaccination and health institutions; the “convenience”, i.e., the ease with which vaccination can be accessed; and “complacency”, that is, from the low perception of the risk of disease [12]. The survey we conducted was not aimed at investigating the “convenience” aspect, as in Italy, access to anti-COVID vaccination is quite simple, widespread and free of charge. Most of the questions were instead addressed to investigate “trust”, particularly through the questions then used for the construction of the Information Score on vaccines in pediatric age, the Information Score of anti-COVID vaccines and the Fear Score; the “complacency” through the questions used to build the Information Score of the pandemic. Most of the compilations of the questionnaire were carried out by women from Northern Italy, especially from the province of Modena: this aspect was predictable, since in Italian culture it is above all the mother who takes care of the health situation of the children. 

The first result worthy of relevance is given by the fact that about 10% of parents who responded to the survey declared that they did not respect the vaccination schedule for their children, a figure in line with what is already known for the Italian population [34]. In the Hesitant/Reluctant group this proportion increased to 22%, to which it is plausible to add a further 23% of “no answer”, while, among the Doubtful, about 0.9% experienced vaccination hesitancy in the pre-pandemic era, to which 3% of those who did not respond can be added. In the group of those who are Favorable, however, the phenomenon of vaccine parental hesitancy in the pre-pandemic era is almost non-existent. Vaccination hesitancy for adult COVID vaccine is highly prevalent in the Hesitant/Reluctant group (only 21% of the group reported having taken or booked the COVID vaccination for themselves, while 99.6% of the enthusiasts and 96% of the doubters entered the vaccination circuit). Our cluster analysis, including questions on “traditional” vaccines and anti-COVID vaccines, should have identified those parents who have had no problems accepting the previous vaccines but have doubts about the new ones. The fact that the "Doubtful" ones were closer to the “Hesitant/Reluctant” is a result to be taken into consideration. Even if not considered “Hesitant” when vaccinating their children, the “Doubtful” parents probably still had doubts or were already sensitive to certain issues. On the other hand, interventions to increase vaccination coverage are done towards the overall coverage and not only towards a single vaccination because it is known that those that are in favor of vaccines usually follow recommendations on vaccination schedule reported by the health authorities of their country [36,37]. Vaccinations are included among public health strategies, and the vaccine plans must be followed in their entirety.

The descriptive analysis of the population and the univariate multinomial logistic regression analysis have identified that vaccination hesitancy against pre-pandemic pediatric vaccines was associated with parental hesitancy/reluctance of anti-COVID vaccination. Among the demographic characteristics, it was noted that the “Doubtful” ones did not differ significantly from the “Favorable” ones, while the “Hesitant/Reluctant” were more likely high-school graduates and less likely with a specialist/master’s degree, were more likely freelancers; in the Hesitant/Reluctant group, there was also a more pronounced propensity not to answer questions.

The Information Scores were all significantly lower for the Doubtful and the Hesitant/Reluctant than the favorable group, both in the univariate and multivariate analyzes. Furthermore, the Fear Score was found to be significantly correlated to the risk of “doubt” and “hesitancy/reluctance”. No association was found between the sources of information and the risk of “doubt” or “hesitancy/reluctance” in the multivariate analyzes (data not shown), although a tendency towards distrust of healthcare personnel was found from the descriptive analyzes. These results are essentially in line with the previous literature on vaccine parental hesitancy of pediatric routine vaccines: it is in fact the distrust of institutions that is one of the main drivers towards vaccinations, in addition to concerns related to the safety of vaccines [26,38,39].

In comparison to previous research studies [27,28,29,30,31], our study has the advantage that it was performed when vaccines against COVID were authorized for use in pediatric age and the public debate on this topic was polarized. Our results describe a real-life situation in an epidemiological context where the discussion on the subject was in the media every day. In addition, another advantage is that we analyzed, in detail, the overall vaccine hesitancy towards vaccinations, showing that a low confidence on vaccines in general has an impact on anti-COVID vaccine acceptance. In the context of anti-COVID vaccination, it is important to note how the spread of the COVID-19 narrative as a disease almost exclusively of the elderly and the frail had probably increased the “complacency” of young people. From the results of the survey, it was found that the younger age (i.e., under 40 years) was more often associated with “doubt” and hesitancy/reluctance. This result appeared in line with the findings of the OCEAN-III trial conducted in the United Kingdom, in which several information notes were produced and administered to homogeneous groups of users: Freeman and colleagues observed that only information that highlighted individual positive aspects anti-COVID vaccination changed the propensity to vaccinate in the “Hesitant/Reluctant” group [40].

Therefore, if we were to draw an identikit of the Hesitant/Reluctant parent towards anti-COVID vaccines, we could identify some characteristics: young parent, with a secondary school or three-year degree, freelance, with a family income below €28,000 and with an erroneous perception of the risk of disease and anti-COVID vaccination. Moreover, the same factors were associated with doubts about anti-COVID vaccination, to which the female gender is added. These results are in line with other surveys aimed at adult vaccination hesitancy, performed both in the Italian [27,41] and the international settings [42,43,44,45]. However, to the best of our knowledge, ours is one of the first surveys conducted during the COVID-19 vaccination campaign after the authorization of anti-COVID vaccines for the pediatric population aimed to comprehensively investigate parental vaccine hesitancy related to the risk perceptions of pediatric vaccines, of COVID-19 and of anti-COVID vaccines in Italy. Our results underlined the importance of correct information on impact of COVID-19 addressed to parents to reduce the risk of parental vaccine hesitancy.

The main limitation of the study is related to the type of survey: anonymous, online and without sampling. In fact, there was an important imbalance of gender and geographical distribution. Moreover, the response rate was not very high (<50%), and this was probably because the questionnaire, consisting of 61 items, discouraged most of the people who accessed the link from completing the survey. However, this aspect is also a strength, as most of the users who completed the questionnaire gave plausible and analyzable answers.

## 5. Conclusions

The aspects related to parental vaccine hesitancy towards anti-COVID vaccines detected by our survey appeared to substantially overlap with the already known factors of parental vaccine hesitancy for routine vaccines: poor ability to correctly perceive the risk of the disease, low-quality level of information on vaccines and on the disease, and generally lower level of education [26,46]. These results, which should be confirmed in larger population and in different geographical areas, should lead institutions and stakeholders to identify targeted communication tools to improve trust in health institutions, especially by younger people. Further studies are needed to identify different communication strategies for different types of vaccination hesitancy [47], such as to improve the level of information regarding the COVID-19 disease and anti-COVID vaccines, to reach the objective of the increased adherence to the mass anti-COVID vaccination campaign for adolescents. Considering that most of the doubters and Hesitant/Reluctant reported having completed the vaccination process of their children according to the recommended vaccination schedule, it would seem appropriate to organize targeted communication campaigns in which the actors are the same who normally promote the vaccinations included in the National Vaccine Prevention Plan [48]: primary-care pediatricians, general practitioners and vaccination centers’ healthcare workers. Given the importance of the role of health professionals [49], it is finally appropriate to identify information and communication-training strategies specifically addressed to them, especially when new vaccines are authorized in the market. This will be important when anti-COVID vaccines are also authorized for children under the age of 12, due to the frequency of asymptomatic SARS-CoV-2 carriers among young children [50,51].

## Figures and Tables

**Table 1:** Comparison of the demographic declared characteristics between the groups.

Demographic Characteristics	Favorable	Doubtful	Hesitant/Reluctant	*p*-Value
Age				<0.001
<40 years	36 (7.55%)	58 (11.03%)	192 (24.12%)	
41–50 years	316 (66.25%)	352 (66.92%)	435 (54.65%)	
>50 years	125 (26.21%)	116 (22.05%)	169 (21.23%)	
Gender				<0.001
Female	381 (79.87%)	455 (86.50%)	467 (58.67%)	
Male	95 (19.92%)	66 (12.55%)	263 (33.04%)	
No answer/non-binary	1 (0.21%)	5 (0.95%)	66 (8.29%)	
Education				<0.001 *
Lower secondary school	19 (3.98%)	40 (7.60%)	29 (3.64%)	
High school	175 (36.69%)	227 (43.16%)	302 (37.94%)	
BA	39 (8.18%)	36 (6.84%)	101 (12.69%)	
MA	171 (35.85%)	161 (30.61%)	210 (26.38%)	
PhD	63 (13.21%)	49 (9.32%)	110 (13.82%)	
No answer	10 (2.10%)	13 (2.47%)	44 (5.53%)	
Work condition				<0.001
Permanent employee	21 (4.40%)	41 (7.79%)	32 (4.02%)	
Temporary employee	312 (65.41%)	352 (66.92%)	380 (47.74%)	
Self-employed	80 (16.77%)	74 (14.07%)	221 (27.76%)	
Unemployed/unpaid work	52 (10.90%)	35 (6.65%)	33 (4.15%)	
Retired	1 (0.21%)	0 (0)	7 (0.88%)	
No answer	11 (2.31%)	24 (4.56%)	123 (15.45%)	
Annual family income				<0.001
<15,000€	17 (3.56%)	23 (4.37%)	56 (7.04%)	
15,001–28,000€	84 (17.61%)	105 (19.96%)	159 (19.97%)	
28,001–55,000€	178 (37.32%)	176 (33.46%)	189 (23.74%)	
55,001–75,000€	69 (14.47%)	61 (11.60%)	83 (10.43%)	
>75,000€	77 (16.14%)	54 (10.27%)	75 (9.42%)	
No answer	52 (10.90%)	107 (20.34%)	234 (29.40%)	
Number of children				<0.001 *
1	98 (20.55%)	125 (23.76%)	242 (30.40%)	
2	276 (57.86%)	300 (57.03%)	379 (47.61%)	
>2	103 (21.59%)	101 (19.20%)	175 (21.98%)	
Number of children 12–17 years old				<0.001 *
1	353 (74.00%)	407 (77.38%)	536 (67.34%)	
>1	124 (26.00%)	119 (22.62%)	260 (32.66%)	
Nationality				<0.001 *
Italy	461 (96.65%)	506 (96.20%)	753 (94.60%)	
Foreign	15 (3.14%)	18 (3.42%)	5 (0.63%)	
No answer	1 (0.21%)	2 (0.38%)	38 (4.77%)	
Area of residence				<0.001 *
North	381 (79.87%)	443 (84.22%)	567 (71.23%)	
Center	58 (12.16%)	53 (10.08%)	94 (11.81%)	
South and Islands	29 (6.08%)	20 (3.80%)	62 (7.79%)	
No answer	9 (1.89%)	10 (1.90%)	73 (9.17%)	

* After Bonferroni’s adjustment: *p* > 0.05 of comparison between Favorable and Doubtful; *p* < 0.05 between Hesitant/Reluctant and other groups.

**Table 2:** Univariate multinomial logistic regression analysis.

Independent Variables	Favorable	Doubtful		Hesitant/Reluctant	
		RR	95% CI	RR	95% CI
Age	1 (Ref.)				
<40 years 41–50 years >50 years		1 (Ref.) 0.69 0.57	- 0.44–1.07 0.35–0.94	1 (Ref.) 0.26 0.25	- 0.17–0.38 0.16–0.38
Gender	1 (Ref.)				
Female Male No answer/non-binary		1 (Ref.) 0.58 4.18	- 0.41–0.81 0.48–36.00	1(Ref.) 2.25 53.84	- 1.72–2.96 7.44–389.75
Education	1 (Ref.)				
Lower secondary school High school BA MA PhD No answer		1.62 1 (Ref.) 0.71 0.72 0.60 1.00	0.91–2.90 - 0.43–1.16 0.54–0.97 0.39–0.91 0.42–2.34	0.88 1 (Ref.) 1.50 0.71 1.01 2.55	0.48–1.62 - 0.99–2.27 0.54–0.93 0.70–1.45 1.25–5.19
Work condition	1 (Ref.)				
Employed Self-employed Unemployed/unpaid Retired/no answer		1 (Ref.) 0.78 0.57 1.69	- 0.55–1.57 0.36–0.89 0.83–3.44	1 (Ref.) 2.23 0.51 8.75	- 1.66–2.99 0.32–0.81 4.76–16.09
Annual income	1 (Ref.)				
<15,000€ 15,001–28,000€ 28,001–55,000€ 55,001–75,000€ >75,000€ No answer		1.36 1.26 1 (Ref.) 0.89 0.71 2.08	0.70–2.65 0.88–1.80 - 0.59–1.34 0.47–1.06 1.41–3.08	3.10 1.78 1 (Ref.) 1.13 0.92 4.24	1.73–5.54 1.27–2.49 - 0.77–1.65 0.63–1.34 2.94–6.09
Number of children	1 (Ref.)				
1 2 >2		1 (Ref.) 0.85 0.77	- 0.62–1.16 0.52–1.12	1 (Ref.) 0.55 0.69	- 0.42–0.73 0.49–0.96
Children 12–17 yrs	1 (Ref.)				
1 >1		1 (Ref.) 0.83	- 0.62–1.11	1 (Ref.) 1.38	- 1.07–1.78
Nationality	1 (Ref.)				
Italy Foreign No answer		1 (Ref.) 1.09 1.82	- 0.54–2.19 0.16–20.16	1 (Ref.) 0.20 23.26	- 0.07–0.56 3.18–170.01
Area of residence	1 (Ref.)				
North Center South and Islands No answer		1 (Ref.) 0.78 0.59 0.95	- 0.53–1.17 0.33–1.06 0.38–2.37	1 (Ref.) 1.09 1.43 5.45	- 0.76–1.55 0.91–2.27 2.69–11.02
Hesitant pre-pandemic	1 (Ref.)				
Yes No answer		1.56 14.98	0.37–6.56 1.98–113.43	64.60 204.73	20.48–203.72 28.56–1467.35
Loved person w/positive swab	1 (Ref.)				
Yes No answer		1.06 0.94	0.82–1.37 0.18–4.71	1.19 17.31	0.94–1.52 5.37–55.80
Loved person w/symptoms	1 (Ref.)				
Yes No answer		1.03 2.76	0.80–1.32 0.28–26.77	0.91 38.58	0.72–1.15 5.31–279.90
Loved person died	1 (Ref.)				
Yes No answer		1.04 5.53	0.76–1.41 0.66–46.18	0.69 35.79	0.51–0.94 4.94–259.40
Inf. score on vaccines (pre-pandemic)	1 (Ref.)	0.05	0.02–0.11	0.004	0.002–0.01
Inf. score on COVID-19	1 (Ref.)	0.42	0.30–0.59	0.01	0.01–0.02
Inf. score on anti-COVID vaccines	1 (Ref.)	0.03	0.02–0.05	0.001	0.0004–0.0013
Fear Score	1 (Ref.)	7.77	5.56–10.87	42.29	29.58–60.45

CI, confidence interval; RR, relative risk; Ref., reference variable.

**Table 3:** Multivariate multinomial logistic regression, identified by comparison of the Akaike information criterion (AIC).

Independent Variables	Favorable	Doubtful		Hesitant/Reluctant	
		RR	95% CI	RR	95% CI
Age	1 (Ref.)				
<40 years 41–50 years >50 years		1 (Ref.) 0.64 0.54	- 0.39–1.04 0.31–0.93	1 (Ref.) 0.30 0.14	- 0.17–0.55 0.07–0.29
Gender	1 (Ref.)				
Female Male No answer/non-binary		1 (Ref.) 0.65 0.62	- 0.44–0.97 0.06–6.21	1 (Ref.) 0.94 0.80	- 0.57–1.55 0.08–8.00
Work condition	1 (Ref.)				
Employed Self-employed Unemployed/unpaid Retired/no answer		1 (Ref.) 0.85 0.44 1.47	- 0.57–1.26 0.26–0.74 0.68–3.17	1 (Ref.) 1.62 0.31 3.29	- 1.01–2.60 0.14–0.67 1.42–7.65
Information Score on vaccines (pre-pandemic)	1 (Ref.)	0.15	0.06–0.34	0.04	0.02–0.10
Information Score on COVID-19	1 (Ref.)	0.46	0.31–0.69	0.05	0.03–0.07
Fear Score	1 (Ref.)	7.05	4.88–10.18	11.43	7.47–17.51

CI, confidence interval; RR, relative risk; Ref. Reference variable.

## Data Availability

All the available data are included in the manuscript execpt the AIC post-hoc analyses for multivariable regressions.

## Appendix A

Questionnaire of the survey.

**Demographic Data**	
Q1	Age
Q2	Gender
	M F Non-binary I prefer not to answer
Q3	CAP (residence)
Q4	Education
	Lower secondary school diploma High school diploma BA MA PhD I prefer not to answer
Q5	Work condition
	Permanent employee Temporary employee Self-employed Retired Unemployed—Unpaid domestic worker I prefer not to answer
Q6	Annual family income
	<15.000€ 15.001–28.000€ 28.001–55.000€ 55.001–75.000€ >75.000€ I prefer not to answer
Q7	Number of children
Q8	Number of children between 12 and 17 of age
Q9	Nationality
	Italy Western Europe Eastern Europe North Africa Central Africa Asia, Americas I prefer not to answer)
Data on pre-pandemic vaccination hesitation	
Q10	Think back to recommended or mandatory pediatric vaccinations: how much do you agree with the following statements? Cast a vote from 1 (disagree) to 5 (fully agree)
Q10.S1	Pediatric vaccines are useful for preventing life-threatening diseases
Q10.S2	Pediatric vaccines have saved millions of lives since they were invented
Q10.S3	Pediatric vaccines are poorly studied
Q10.S4	Pediatric vaccines often lead to serious adverse events
Q10.S5	Pediatric vaccines are safe
Q10.S6	Vaccinating children is important to stop some epidemics (e.g. measles, rubella, whooping cough, meningococcal meningitis)
Q11	We ask you to rethink your experience with your children’s pediatric vaccination process: how much do you agree with the following statements? Cast a vote from 1 (disagree) to 5 (fully agree)
Q11.S1	I appreciate it when I get invitations for my kids’ vaccinations
Q11.S2	When I receive invitations for vaccinations, I start to feel anxious
Q11.S3	I trust the institutions, the proposed vaccines are the best choice for my children
Q11.S4	I do not trust what is proposed, there is not enough information
Q11.S5	The information that comes from the AUSL or from the institutions is partial or incorrect
Q11.S6	After vaccination, I am afraid of serious adverse events
Q11.S7	I consult my doctor and decide on the basis of his advice
Q12	Did all of your children have pediatric vaccinations according to the recommended schedule?
	No, because I didn’t have enough reassurance Yes No but for delays due to health reasons I prefer not to answer
Data on Covid-19 risk perception	
Q13	We ask you to rethink your experience throughout the pandemic period, from March 2020 to today: how much do you agree with the following statements? Cast a vote from 1 (disagree) to 5 (fully agree)
Q13.S1	The pandemic has been magnified by governments and the media
Q13.S2	Covid-19 is a normal flu or a little more
Q13.S3	Covid-19 scared/scares me
Q13.S4	The pandemic has deteriorated the possibilities of prevention and treatment by the Health System
Q13.S5	Long-Covid is a risk that I would not like to take
Q13.S6	It is easy to avoid the virus that causes Covid-19 disease
Q13.S7	Children and young people, even if they get sick, never have problems
Q13.S8	The disease is dangerous only for elderly people and those with pathologies
Q14	Have you or a family member or loved one had Covid-19 (positive swab)?
	Yes No I prefer not to answer
Q15	Have you or a family member or loved one had symptoms or have you been hospitalized for Covid-19?
	Yes No I prefer not to answer
Q16	Has a family member or loved one died due to Covid-19?
	Yes No I prefer not to answer
Data on perception of the anti-Covid vaccination	
Q17	We now ask you to rethink the information you have received on anti-Covid vaccines: how much do you agree with the following statements? Cast a vote from 1 (disagree) to 5 (fully agree)
Q17.S1	Authorized vaccines are still experimental
Q17.S2	Many dangerous health effects of vaccines are unknown
Q17.S3	Vaccines are safe for me
Q17.S4	Vaccines are useless to contain the spread of the virus
Q17.S5	Vaccines will give rise to dangerous variants of the virus
Q17.S6	Vaccines are produced in ways that I cannot accept for religious reasons
Q17.S7	Vaccines are the best way to avoid deaths and hospitalizations
Q17.S8	mRNA vaccines (Pfizer and Moderna) are safe, adenoviral vector vaccines (AstraZeneca and J&J) are not
Q18	What are your main sources of information on vaccines? Express a vote from 1 (“Not authoritative”) to 5 (“Most authoritative source”), express 0 for “Never consulted”
Q18.S1	Family Doctor or Pediatrician
Q18.S2	Doctor / s within the National Health System (Public Hygiene Service, Community Pediatrics, hospital doctors)
Q18.S3	Traditional TV and media (newspapers, magazines) or social network sites/channels connected to them
Q18.S4	Religious personalities (priests, imams, rabbis...)
Q18.S5	Social networks (Facebook groups, Twitter, Instagram, Whatsapp, Telegram…)
Q18.S6	Social dissemination profiles (university professors, researchers or popularizers such as, by way of example only: Roberta Villa, Roberto Burioni, Guido Silvestri, Antonella Viola)
Q18.S7	Friends/acquaintances
Q18.S8	Websites and/or institutional social channels (ISS, AIFA, Ministry of Health, World Health Organization, ECDC...)
Q18.S9	Counter information websites
Q18.S10	Independent doctors
Q19	Have you done or booked the anti-Covid vaccination?
	Yes No I prefer not to answer
Q20	Would you recommend anti-Covid vaccination to your friends/acquaintances?
	Yes No I prefer not to answer
Data on perception of anti-Covid vaccination in adolescents of 12–17 year of age	
Q21	We ask you to focus now on the possibility of vaccinating your children between 12 and 17 years old: how much do you agree with the following statements? Cast a vote from 1 (disagree) to 5 (fully agree)
Q21.S1	I have vaccinated/I will definitely have my children vaccinated
Q21.S2	I am not going to vaccinate my kids
Q21.S3	The vaccine has an excellent level of safety
Q21.S4	The vaccine has not been sufficiently tested
Q21.S5	I want to wait before I vaccinate my children
Q21.S6	My children are healthy, they don’t need vaccines for them
Q21.S7	I have vaccinated/will vaccinate my children because they are vulnerable due to pathological conditions
